# The geography of the age at menopause in central Portugal since the early twentieth century

**DOI:** 10.1038/s41598-022-25475-w

**Published:** 2022-12-20

**Authors:** Rui Martins, Bruno de Sousa, Vítor Rodrigues

**Affiliations:** 1 Departamento de Estatística e Investigação Operacional, Faculdade de Ciências, Universidade de Lisboa, Lisboa, Portugal; 2 Centro de Estatística e Aplicações da Universidade de Lisboa (CEAUL), Lisboa, Portugal; 3 Faculty of Psychology and Education Sciences (FPCE), Center for Research in Neuropsychology and Cognitive and Behavioral Intervention (CINEICC), University of Coimbra, Coimbra, Portugal; 4 Faculty of Medicine, University of Coimbra, Rua Larga, 3004-504 Coimbra, Portugal; 5 Liga Portuguesa Contra o Cancro, Núcleo Regional do Centro, Rua Dr. Antonio José de Almeida, 329 – piso 2 - Sala 56, 3000-045 Coimbra, Portugal

**Keywords:** Social evolution, Risk factors

## Abstract

This work aims at studying the spatio-temporal evolution of the age at menopause in central Portugal since the early twentieth century. We analyzed $N=320,444$ women that had already reached the menopause within a free breast cancer screening program between 1990 and 2018 and born in the period 1910–1960. One of the concerns was about early or late menopause thus we considered percentile regression to build the respective percentile curves inside the package GAMLSS in R. In order to capture the correlation at the regional level, a spatial random-effect was considered. The obtained clustered spatial effects were analyzed to assess geographical differences among the percentiles of the age at menopause by year of birth. An increasing trend in the median age at menopause and regional differences for all the considered percentiles were found. From 47.1 years in 1910 to 49.59 years in 1960 (about 2.49 years in 5 decades). Early and premature menopause (below percentile 5%) occur in the interior north (north-eastern). Late menopause (above percentile 95%) occur predominantly in the central-north and central-south areas.

## Introduction

Menopause, a term to denote the cessation of menstruations, signs a major life transition with profound effects on a woman’s health. Apart from being a biological threshold for human reproduction, it seems to have a really tight biological-age interval in which it must occur. Women having their menopause outside that interval have an increased risk for several health problems, namely type 2 diabetes^1^ and some types of cancer. Later Age at Menopause (AaM) has been associated with an increased risk of breast cancer^2^ and an early onset of menopause associated with an increased risk of several other diseases such as: cardiovascular^3^, neurological, psychiatric, osteoporosis, and other sequelae^4^. On the other hand, longer overall survival and greater life expectancy are associated with later AaM^5,6^.

Menopause is influenced by biological, environmental, socio-demographic and lifestyle factors and may be delayed for a woman that had an early menarche, longer and regular menstrual cycles, use of contraceptive hormones, had multiple births and breastfed^7–10^. In contrast, it may be accelerated by residence in an urban community^11^, smoking, over weight, alcohol consumption, malnutrition during infancy or adolescence, poor educational level, living more than 3000 m above sea level and, in general, living in underdeveloped countries^12,13^.

In ancient age some reports talk about the onset of menopause around 40 years old. Estimates for the middle age until the nineteenth century point to an AaM of about 45 years old^14^. Nowadays a mean AaM lower than 48 years has been found only in a few populations (e.g. Philippines, New Guinea^15^ and Mexico^7^). According to two studies^16,17^ involving women aged 45 and older in the 80’s and 90’s of the 20th century, in western countries, the median AaM has been observed to vary between 50 and 52 years old. The European Prospective Investigation into Cancer & Nutrition (EPIC) study found that mean AaM between 1991 and 2007 in 26 centers from 8 of 10 EPIC countries, was 48.6 years, and only 4.8% of women had a premature menopause^1^, i.e. before 40 years of age. An early menopause refers to menopause that occurs at or before age 45, below the median age of natural menopause (age 51 years)^4^.

The Decisions at Menopause Study (DAMES)^18–20^ conducted in Lebanon, Morocco, Spain and USA, with women aged 45–55 born in the period 1940 to early 1950’s, estimated that the median AaM were, respectively, 49.3, 48.4, 51.7 and 52.6. In a cross-sectional survey across Poland in 2000–2004 with women aged between 35 and 65, the overall median AaM was found to be 51.25 years; the 25th percentile was 49 years and the 75th percentile was 54 years^21^.

Contrary to what happens with the age at menarche, the AaM is not so well documented. There is a lack of research on its temporal trends. Some point to an increasing age worldwide^22,23^. Others, however, report that there is no conclusive evidence for such an increase^15,24,25^. We did not find any study suggesting a negative trend in AaM. The study^26^ of the Chuvasha population in a rural region of the Russian Federation point to a mean value of AaM increasing from 47.0 (in women born during 1920–1925) to 49.7 (women born during 1940–1945) and 49.3 (born during 1945–1950). An increase in the average AaM was also observed for a population of women residents of New Hampshire, Massachusetts, USA, born between 1910 and 1969, from 49.1 years in the 1915–1919 cohort to 50.5 years in the 1935–1939 birth cohort^27^.

Official statistics show that Portugal is one of the most aged countries in the world^28,29^, i.e. with one of the highest percentages of older people per new born, thus the concerns with the ageing of the population and well being and clinical consequences associated with menopause highlight the importance of research designed to identify age variation in natural menopause and factors predicting women’s transition to menopause in order to have better public health policies^30^. The Portuguese panorama is identical to those already described above. A work^31^ that investigates the reproductive period and fertility in a rural Portuguese municipality (Oleiros) claims that the mean AaM for that municipality was 48.69 years for a woman born in 1880–1940. Despite being focused on a specific-municipality that work has the advantage, for our study, of analyzing a municipality that is part of our own study, during a time-period that precedes the time period analyzed in this work and is likely to have some overlapping women.

A typical regression analysis, in most contexts, focuses on explaining the expected value dependency of a response variable as a function of a set of explanatory variables. Although in some situations, if we want to understand not only the behavior of the average person, but also the behavior of those who belong to the extremes of the population, we might consider percentile regression^32,33^ that allows us to go beyond mean regression, enabling building regression curves for the percentiles, instead of the mean of a response variable. Since the starting point for this work was the idea of understanding the spatio-temporal evolution of menopause in the central region of Portugal and considering the potential impact of early and/or late menopause on a women’s health, this study will analyse a dataset from the Breast Cancer Screening Program described in the section Data Description below, considering a distributional regression approach. As Rigby et al.^34^ argue, for data with more than 1000 observations, regression models beyond the mean should be the norm, not the exception.

## Data description

The study of the AaM percentiles in the central region of Portugal was carried out considering a dataset on the Breast Cancer Screening Program provided by the Portuguese Cancer League (LPCC) in the Central region of Portugal in the period 1990–2018 which has screened 452,342 women born in the period 1900–1973. At the age of 45, or 50 after 2010, all women in each of the 89 municipalities (see Fig. 3) are invited to have a free screening mammogram and every 2 years thereafter until the age of 69. This region roughly represents 25% of the Portuguese population. More details about the screening program and the inclusion criteria are given elsewhere^35–37^. Age at menopause, relying solely on the correctness of a woman’s memory, was defined as the self-reported age at the last menstrual period and no information was given on whether menopause was naturally occurring or induced. We stress that as women are screened several times during their life about their AaM, and given that sometimes the answer does not coincide with the last ones, for purposes of analysis we consider the most frequent answer (age) given by that particular woman.

For statistical analysis purposes we decided to withdrawn women born before 1910 because they were only 139, which is considered to be a small sample size for a centile regression. We also decided not to analyze the AaM of women born between 1961 and 1973 because this cohort is still quite incomplete, with a very high percentage of women who have not yet reported having reached menopause. Furthermore, an initial analysis of the database with these women included revealed some inconsistencies in the results. Additionally, due to missing values for the AaM we will be considering only the complete cases for the period 1910–1960, i.e. we exclude all the women without an observed menopause. Obviously, we are aware of the inherent statistical problems of such truncation of this dataset, because this might lead to biased results, as we argue in the “Discussion” section at the end of the article. Namely: (i) women removed from the calculations are mostly the youngest and they might have a different life story (i.e. influencing covariates) than the oldest, what could be reflected in their, possible, different menopause timings; and (ii) women having the most recent menopause observed are young with early or even premature menopause. These two characteristics have the potential of shrinking the estimates for the AaM towards lower values. Four women were also removed because they reported a menopause age above 69, which is considered unrealistic^27^, and one girl due to have reported a menopause age below 15. A premature ovarian insufficiency affects 10 girls per 100,000 person-years for girls of 15–29 years of age^38^.

**Figure 1:**
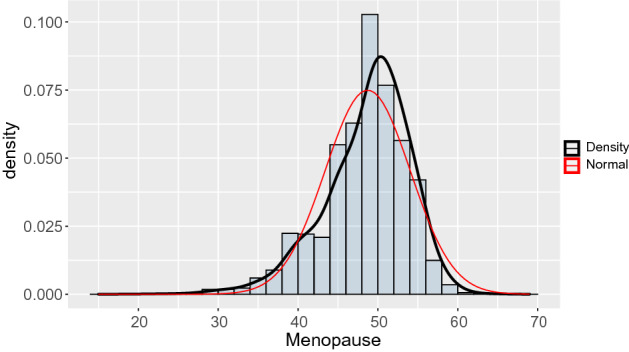
Age at menopause. The variable has a left (negative) skewed distribution.

In the end we were left with a sample size of $N=320,444$ women who have reached the menopause. Age at menopause ($\texttt {AaM}$) registered in years at the first interview, was the response variable of interest. The average was 48.72 with a minimum of 15 and a maximum of 69. Figure 1 shows the histogram for the unconditional distribution of this variable with a modal class at 48–50 years old. Summarized in Table 1 are its descriptive percentiles by period since 1910 to 1960 and Table 4 depicts the mean and the median by municipality ($\texttt {Muni}$) and birth year ($\texttt {Byear}$). In Table 5 we present the percentage of women with an observed menopause per municipality since 1910 to 1960 in our data set.

**Table 1:** Summary of the AaM percentiles by period of birth since 1910 to 1960.

Age at menopause (Percentiles)								
Birth Period	5	10	25	50	75	90	95	Women
10–20	38	40	45	49	51	53	55	3466
21–30	38	40	45	49	52	54	55	22953
31–40	38	40	45	49	52	55	55	77607
41–50	39	42	45	50	52	55	56	108563
51–60	40	42	47	50	53	55	56	107855

Last column presents the total number of women screened.

## Methods

A typical regression analysis has the advantage of being very well know to the users, although it is very likely that in many scenarios other properties of the response distribution (e.g. the variance) may also depend on covariates. Besides this, one may want to comprehend, not only the behavior of the average person, but the behaviour of those that belong to the population’s extremes. To account for these features, we will consider a statistical framework developed within the context of Generalized Additive Models for Location, Scale and Shape (GAMLSS)^39^, also known as distributional regression models^40^. These models have several advantages, but for our work the principal characteristic is that it assumes a known parametric family of distributions for the response variable which allows an easy calculation of the conditional percentiles curves of the AaM given the birth year and the municipality of residence. Additionally, the effects of covariates that we are interested in can have flexible forms (e.g. smoothing functions) not being restricted to the traditional, and several times unrealistic, linear effect. At the same time it ensures that the adjusted percentile curves do not cross. A competitor method is quantile regression^41^, where the adjusted curves might cross, because it does not assume a distribution for the response variable and, therefore, can be considered in the realm of non-parametric methods^34^. Other authors have been considering other approaches. Most use simple statistical methods like ANOVA^42^ or linear regression^26^. Frommer^22^ considers a probit model, Reynolds and Obermeyer^20^ a logit regression and a survival analysis is used by Shanley and Kirkwood^43^ and Kaczmarek^21^.

### Statistical models

Based on data for $i = 1, \ldots , N=320,444$ women, we will assume conditional independence of the individual ages at menopause, AaM, given the covariates Byear and Muni. An initial exploratory analysis showed that the distribution of the AaM is left (negative) skewed (*vide* Fig. 1) and a flexible approach based on splines will allow us to capture any (possible) nonlinear effect of the predictor Byear on the AaM. This assumption do not precludes a possible simple linear relationship between those two variables, but it is rather a parsimonious assumption. Taking this into consideration we decided to analyze the data with a model within the aforementioned class—GAMLSS. This method permit an highly flexible approach, because we are no longer constrained to the traditional distributional assumptions, like normality for example, and we are free to utilize skewed distributions without the need of transforming the data, allowing us to work on the scale of the data which is a very important feature. Additionally, as already said, all the parameters of the response probability distribution can be modeled by explanatory variables and not only the location. For instance, it is possible to model the parameters related to the scale and shape (for some distributions it is also possible to model the kurtosis). All the explanatory variables are introduced into the model parameters through predictors, which can be linear functions of the explanatory variables or can take the form of structured additive predictors with non linear or smoothing functions of explanatory variables.

The generalized Akaike Information Criterion (GAIC), a models selection measure, was considered to select the best fitting distribution of the data. It was found that the *Box–Cox Cole and Green* distribution, $\text {BCCG}(\mu ,\sigma ,\nu )$, provides the lower GAIC value (i.e. the best fit) when compared to other alternative distributions. Additionally, it was found that modeling the parameters $\mu$ and $\sigma$ as functions of both the birth year and the municipality also improves the GAIC value (i.e lowering it) when compared to a model where these parameters are independent of those covariates. Taking that into account the main statistical model for analyzing the data within the GAMLSS framework that we will be dealing with is:

1 $$\begin{aligned} \texttt {AaM}&\sim \text {BCCG}(\mu ,\sigma ,\nu ), \nonumber \\ \mu&= \beta _{10} + f_{11}(\texttt {Byear}) + f_{12}(\texttt {Muni}), \nonumber \\ \log {(\sigma )}&= \beta _{20} + f_{21}(\texttt {Byear}) + f_{22}(\texttt {Muni}), \nonumber \\ \nu&= \beta _{30}. \end{aligned}$$

We defined an additive model for the location parameter, $\mu$, which for this distribution represents its median, a very important parameter within our work, as we are interested in estimate the percentiles, so the linear predictor that we defined, $\mu = \beta _{10} + f_{11}(\texttt {Byear}) + f_{12}(\texttt {Muni})$, allows us to directly model the median. For the scale of the response variable, $\sigma$, we let the parameter depend on the explanatory variables Byear and Muni. It is a multiplicative model resulting from the log-link, which in turn ensures positive values for the parameter. For this distribution the scale parameter is approximately the coefficient of variation. The shape parameter, $\nu$, is modelled only with an intercept term. It was found that adding covariates to its linear predictor did not improve the adjustment.

The functions $f_{11}$ and $f_{21}$, modelled as cubic splines, represent the temporal effects of the year of birth, and $f_{12}$ and $f_{22}$ are the spatially correlated effects of the residence municipalities modeled as an intrinsic autoregressive process (IAR), a limiting case of the conditional autoregressive models (CAR) of Besag et al.^44^, because we are considering that the spatial effect is a Markov random field (MRF), i.e. we assume that the spatial random effects (our spatial variables) have a joint distribution which is specified by considering conditional independence locally. The IAR models are a typical choice when dealing with a dependent variable observed in geographical areas sharing borders, because we are expecting neighboring areas to have more similar observed values for the AaM than areas far apart. The consequence of this approach is that the parameter estimates for neighboring locations are shrinked towards its mean.

### Ethics approval

This project has used data from the Breast Cancer Screening data from Portugal, which have ethical approval from the Faculty of Psychology of the University of Coimbra Ethics Committee and the Portuguese Cancer League.

### Consent to participate

Usage of data derived from the records is according to Portuguese and European laws and regulations. All women signed the informed consent prior to the screening procedure.

## Results

The conditional percentile values were thus obtained within the R software^45^, namely using the main package gamlss (version 5.1-4) and two more packages that provide a set of functions to fit models with spatial variables (gamlss.spatial and gamlss.add). Below the chosen model is written in terms of the R syntax based on the gamlss package:
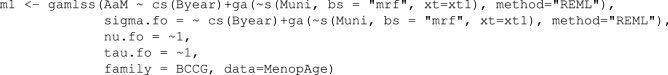
where mrf means Markov Random Field and xt1 conveys the information about the spatial neighborhood structure considered for our application. Specifically, we considered the so-called first-order neighborhood structure, i.e municipalities that share a boundary are considered neighbors in the central region of Portugal.

Model diagnostics were based on the normalized quantile residuals as defined by Ref.^46^, p. 418. A summary of these residuals is listed in Table 2. We can see that, they have a nearly zero mean, a variance nearly one, a coefficient of skewness near zero and a coefficient of kurtosis near 3. These are all typical characteristics when the residuals are approximately normally distributed with zero-mean and variance equal to 1. From here we can conclude that the selected model is adequate.

**Table 2:** Summary of the normalized quantile residuals.

Statistic	Estimate
Mean	0.00055
Variance	0.99584
Coef. of skewness	0.02446
Coef. of kurtosis	3.17719

### Age at menopause—temporal trends

**Figure 2:**
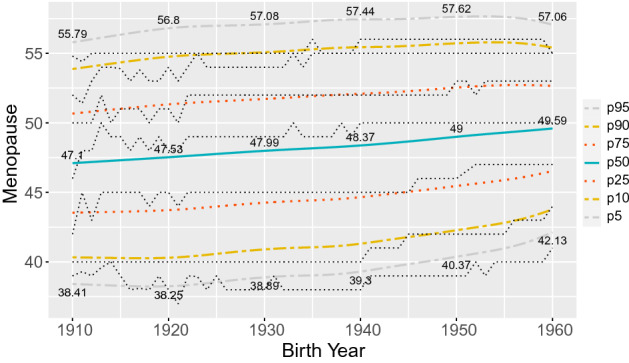
Estimated percentile curves for the age at menopause by year of birth since 1910. The values printed are for the percentiles 5%, 50% and 95% for the years $1910,\ldots ,1960$. Lines with small black dots represent the correspondent observed percentiles.

A first distributional regression model without covariates was considered in order to estimate the median AaM for the overall population since 1910, resulting in a point estimate of 49.38 years old. Then we limited the model to one explanatory variable Byear for exploring the temporal trends in the central region of Portugal in AaM (Fig. 2). It should be noted that the percentiles curves displayed are not linear. This behavior could not be captured within a typical linear regression analysis and is facilitated by the non-linear approach permitted by the generalized additive models. Additionally, the estimated percentiles 5%, 10%, 25% and 50% are steadily increasing at a greater rate since 1920, than the estimated percentiles 75%, 90% and 95%, which could mean that there is no much more space for increases in later menopause ages. Globally, most of the estimated percentile curves fit quite well to the observed data. The exception is the 95% percentile curve which deviates from the observed 95% percentiles about 1.5 years.

### Age at menopause—spatio-temporal trends

**Figure 3:**
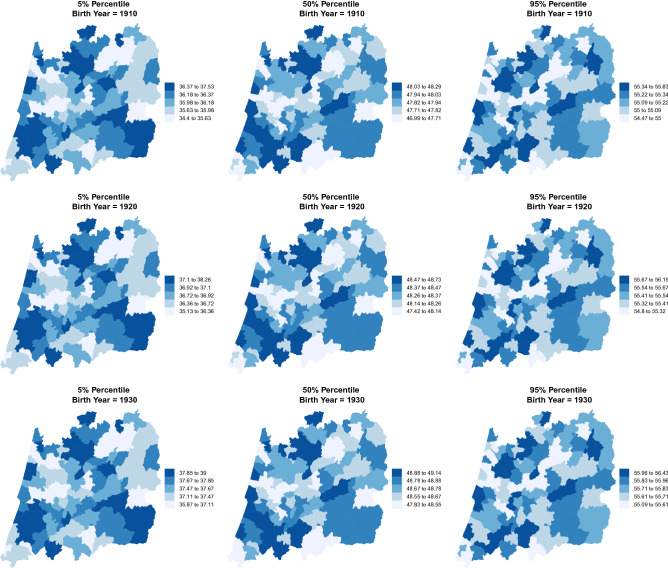
Estimated AaM by region and birth year. Percentiles 5%, 50% and 95% for the central region of Portugal for the years 1910, 1920 and 1930.

**Figure 4:**
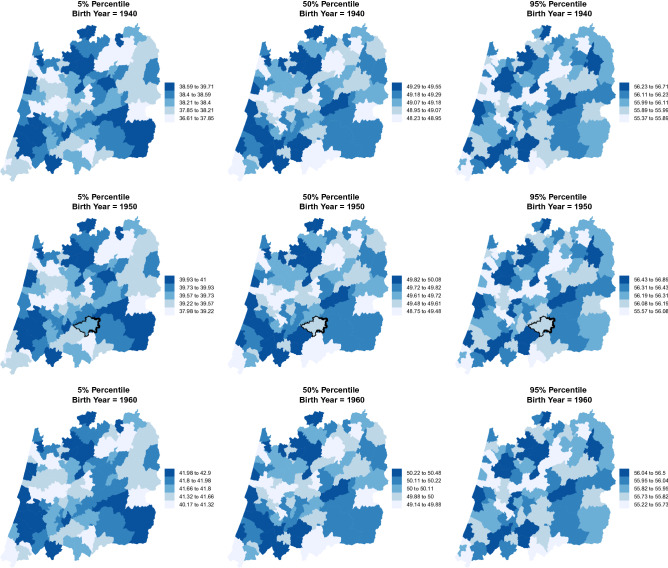
Estimated AaM by region and birth year. Percentiles 5%, 50% and 95% for the central region of Portugal for the years 1940, 1950 and 1960. The highlighted area for the year 1950 represents the municipality of Oleiros.

Each municipality is likely to have the median and the variability of the AaM close to those of its neighbours. Bearing this in mind, spatial effects, $f_{12}(\texttt {Muni})$ and $f_{22}(\texttt {Muni})$, were added to the model in (1) aiming at capturing the influence of neighbouring locations not available by the observed covariates. Figures 3 and 4 present the spatio-temporal trends in the percentiles for the AaM, which are already accounting for the spatial dependency of the response variable (AaM). It is clear that the patterns through the decades have remained unchanged, i.e. regions with the larger values in 1910 maintain its position in 1960, despite all have being increasing, with only a few exceptions.

**Figure 5:**
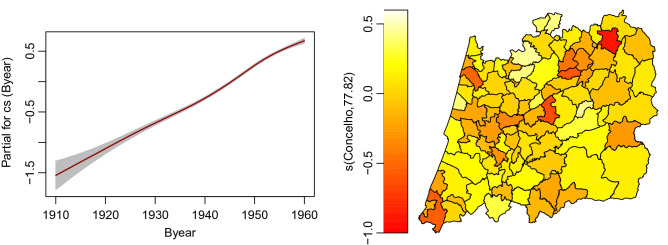
Estimated effects for the age at menopause model showing the temporal variation of the birth year effects (left) together with the clustered spatial effects (right).

Figure 5 depicts the effects of each of the covariates (Byear and Muni) on the AaM location parameter, i.e. its median.

The interpretation is relatively straightforward. An increasing effect of the Byear was found. Additionally, the effect is positive only for those woman born in the period 1942–1960, meaning that a woman born in this period tends to have an AaM above the median for the overall population and accounting all the years, i.e. 49.38. For the spatial effects, a municipality with a negative effect means that women residing there, and controlling for the year of birth, have an AaM below the median when comparing to the overall population. On the other side, areas with a positive spatial effect will tend to have AaM above the median of the overall population. Looking to Figs. 3 and 4 and comparing it to Fig. 5 it is clear that larger values of the median AaM are associated with a positive spatial effect and vice-versa.

Another interesting feature of the temporal effect for the year of birth is obtained considering the getPEF function (Partial Effect function) which allows us to calculate the slope of the curve at each year (*vide* Table 3). For example for the year 1920, in gamlss syntax this can be done by
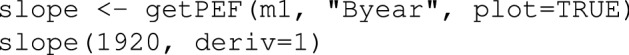
and the result is the slope of the curve’s tangent for the year 1920, which is not very different from the remaining years. The result is approximately 0.044, meaning that for that year the median AaM was increasing at a rate of $0.044 \times 365\approx 16.06$ days, or 5.35 months per decade.

**Table 3:** Estimated increasing rates per year (in days) of the median AaM.

Birth year	Increasing rate (days/year)	Birth year	Increasing rate (days/year)
1910	$0.044 \approx 16.06$	1915	$0.044 \approx 16.06$
1920	$0.044 \approx 16.06$	1925	$0.042 \approx 15.33$
1930	$0.040 \approx 14.06$	1935	$0.039 \approx 14.24$
1940	$0.047 \approx 17.16$	1945	$0.055 \approx 20.08$
1950	$0.055 \approx 20.01$	1955	$0.039 \approx 14.24$
1960	$0.044 \approx 16.06$		

**Figure 6:**
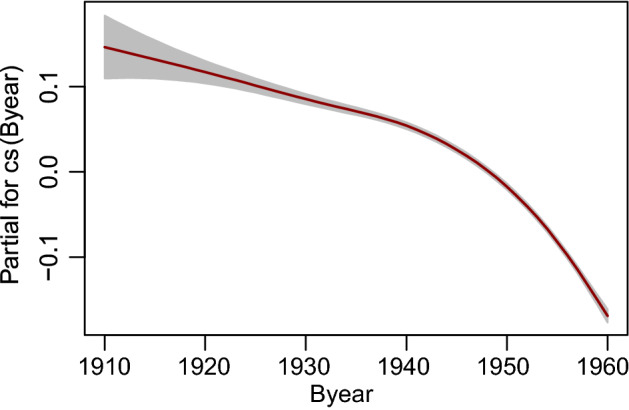
Estimated $\log (\sigma )$ as a function of Byear.

The log-scale parameter, $\log (\sigma )$, shows a decreasing trend as a function of the Byear (Fig. 6), thus larger values of this covariate imply a reduction of the variability in the AaM estimates.

## Discussion

The dataset on menopause here analyzed is based on the largest sample size reported until the date in Portugal, although, and for obvious reasons, it lacks women born in the last decades, and because of that we can not ascertain the percentiles for those youngest women. Yet, and to the best of our knowledge, this work is the first in analyzing and producing percentiles curves for the women residing in the center of Portugal since 1910 and in using a distributional regression approach. Indeed, the time of follow-up of each woman, which can goes up to 24 years, and the time-period, nearly 30 years (1990–2018) are also noticeable. As pointed in Ref.^23^ to study a secular trend in AaM one must have a large population followed over a period of 30 or more years.

Menopause is an imperfect measure of what is, essentially, a long transition noted in retrospective, of something familiar, until the cessation. As mentioned by Sievert^47^, perhaps it is best to only trust the responses of women aged 60 years or younger, because assessment of AaM is based on self-report, which is prone to recall bias, particularly in older women. However, other studies have shown that the validity and reproducibility of self-reported AaM are fairly good^48^. Our study fulfills this point of view, because all women start to be asked whether or not they already reached the menopause by the age of 45. Sievert^47^ also argues that median AaM, as computed by probit analysis, is the only measure of central tendency that does not rely on memory. In this regard we consider that our distributional regression approach has several advantages and improvements over that more traditional and somehow outdated technique.

We resorted in a distributional regression model to estimate several spatio-temporal percentiles for the AaM in the central region of Portugal considering a long period of time and using a representative sample with a huge number of observations. Results show that, within the period 1910–1960, most percentiles obtained for the AaM have an increasing trend. For a girl born in 1920 we would expect a median AaM around 47.53 years, and for a girl born in 1960 that value is greater by about 2 years (Fig. 2), excluding any effects of other covariates. In the middle of the 20th century the increasing rate achieved its maximum of around 20 days per year (Table 3). Interesting, is the pattern of the AaM distribution by municipality. Despite all its percentiles are increasing, the regions with the highest percentile values (respectively, lowest) for AaM in 1920 are the same for 1960 (Figs. 3, 4), with only a few exceptions. Early and premature menopause (below percentile 5%) occur in the interior north (north-eastern) and in a vertical (north to south) band close to the coast, but not containing the municipalities with a sea border (western) of Portugal’s central region for all the decades considered. Later menopause (above percentile 95%) occur predominantly in the central-north and central-south areas.

If we compare our estimate for the municipality of Oleiros with that obtained by Ref.^31^, it becomes clear that AaM is increasing over time. From their estimate of 48.69 for a women born between 1880 to 1920, our estimate for the 1950’s shows an increase from about 1 year to about 49.6. (*vide* Fig. 4 where this area is highlighted.)

The work of Duarte et al.^36^ have already analyzed this data set, but only concerning women who had a mammogram until 2007. In this work we have 11 years of subsequent observations, so a good comparison between both periods is possible. Those authors, in a scenario of complete case analysis, stated that women born after the first world war, are having their menopause at lower ages. With this additional years of data that we have, it is definitely clear that we are facing an increasing trend. In this point our findings agree with Dratva et al.^49^, which claim that AaM is shifting towards higher ages.

We must stress that some of the spatial effects may not being correctly captured, because the spatial information that the dataset enclosures is about the place of living at the time of the participation in the screening program, i.e. when women are adults. Although, we assume that these women have been living there always. This assumption is more likely to be true for the interior areas (east), which have at the same time, lower levels of wealth, than for the areas closest to the coast (highest levels of wealth).

The probability distribution that we chose for describing the AaM, $\text {BCCG}(\mu ,\sigma ,\nu )$, has several advantages for have being considered in this work. First its location parameter, $\mu$, is interpreted as the median of the distribution, which in a work looking for describing the percentiles is a benefit. Additionally, we are free to control the shape parameter, $\nu$, representing the amount of skewness of the distribution and from Fig. 1 we know that our distribution is left skewed. Estimation of the percentiles curves for a response variable is widely used in medicine for checking whether an individual have an abnormally low or high value of the response variable (given the covariates of interest), and hence whether she/he is potentially at risk. For example, a specific country’s region may be the target of differentiated public policies if it is known that girls living there tend to have an early or late menopause. For example, the North American Menopause Society, the British Menopause Society, and the International Menopause Society recommend oestrogen replacement therapy for women with premature or early menopause until at least around the median age of natural menopause (approximately age 51 years)^50–52^.

This work does not consider the possible impact of an adnexectomy in the AaM simply because in the screening program women are not asked about that. Although, and considering that the possible effects of an adnexectomy include an early menopause, the potential impact of those women in the estimated AaM in this work would possibly be in lowering the estimates obtained for the AaM.

Education is known to be, to a certain extent, a surrogate for wealth and lifestyle behaviors, and consequently, has we already said in the introduction, older AaM have been reported to be potentially associated with a higher level of education. Unfortunately, this work did not targeted this relation. During the analyzed period 1910–1960 women in Portugal had mainly domestic and agriculture related works and the population were living mostly in rural areas. There were only 3 years of mandatory schooling but many families, mainly in the countryside, just sent their children to school long enough to be able to read. In 1930 68% of the Portuguese were illiterate. In 1950 only 6.3% of the kids aged 10–11 completed the mandatory 3 years of primary school^53,54^. In any case, the difference between being able to read or not was sufficient to have a job outside the subsistence farming and everything that could come of it. The number of newborns per woman was 3.2 children on average.

In 1960 economic conditions in Portugal were obviously better than in 1910 but not yet similar to the rest of Europe. After world war II the individual income start to grow but slowly, and after 1960, a period not covered in this work, the country experienced a rapid increase of the individual income until the early 70’s^55^. In the coast of Portugal (municipalities in the left of the maps) where the largest cities were concentrated and consequently regions with higher economic conditions, we can see from Figs. 3 and 4 that the AaM is higher than in the interior (municipalities in the right of the maps) where the population mostly lived from the agriculture. Across the country, serious food insecurity problems were faced. In particular, among those who lived in areas whose economic conditions were more unfavorable, as is the case of the central area of Portugal presented here and mainly in its eastern municipalities. This also led to migration to coastal municipalities and emigration, mainly to Brazil, France and Germany (Tables 4, 5).

**Table 4:** Mean $({\bar{x}})$ and median (med) for the AaM by municipality since 1910 to 1960 (Descriptive statistics).

Period (birth year)						Period (birth year)					
Municipality	10–20	21–30	31–40	41–50	51–60	Municipality	10–20	21–30	31–40	41–50	51–60
	${\bar{x}}$; med	${\bar{x}}$; med	${\bar{x}}$; med	${\bar{x}}$; med	${\bar{x}}$; med		${\bar{x}}$; med	${\bar{x}}$; med	${\bar{x}}$; med	${\bar{x}}$; med	${\bar{x}}$; med
Águeda	46.06; 47	47.11; 48	47.91; 49	48.77; 50	49.29; 50	Montemor-o-Velho	NA; NA	49.95; 50	48; 49	48.93; 50	49.45; 50
Aguiar da Beira	46.82; 47	47.66; 50	48.41; 49	48.92; 50	49.61; 50	Mortágua	46.27; 46	47.78; 49	47.69; 49	48.75; 50	49.23; 50
Albergaria-a-Velha	NA	47.99; 49.5	48.61; 50	48.63; 50	49.13; 50	Murtosa	NA	48.65; 50	48.73; 50	49.13; 50	49.59; 50
Alcobaça	48.18; 50	48.08; 50	48.10; 49	48.46; 49	47.50; 48	Nazaré	44.90; 47	46.37; 48	47.53; 49	47.86; 49	46.94; 48
Almeida	47.31; 48.5	48.72; 50	48.49; 50	49.08; 50	49.65; 50	Nelas	45.61; 46	47.54; 49	48.13; 50	48.89; 50	49.51; 50
Alvaiázere	48.31; 50	47.64; 49	47.89; 49	48.73; 50	49.30; 50	Oleiros	46; 47	47.30; 49	48.01; 49	48.51; 49	50.34; 51
Anadia	48.04; 49	47.75; 49	47.92; 49	48.49; 50	49.38; 50	Oliveira de Frades	48.03; 49	47.29; 48	47.97; 49	48.98; 50	49.94; 50
Ansião	47.79; 48	47.11; 49	47.62; 49	48.70; 50	49.10; 50	Oliveira do Bairro	48.05; 50	47.92; 50	47.91; 49	48.44; 50	49.20; 50
Arganil	46.93; 48	48.11; 50	48.34; 50	48.83; 50	49.49; 50	Oliveira do Hospital	50; 50	48.21; 49	47.98; 49	48.81; 50	49.58; 50
Aveiro	NA	48.84; 50	48.46; 50	48.80; 50	49.44; 50	Ourém	NA	48.31; 50	48.34; 49	48.41; 49	47.24; 48
Batalha	49.67; 50	48.05; 49	48.17; 49	48.63; 50	49.83; 50	Ovar	43; 43	47.40; 49	48.58; 50	48.94; 50	49.52; 50
Belmonte	45; 45.5	48.47; 50	48.62; 50	48.70; 50	49.75; 50	Pampilhosa da Serra	NA	NA	48.23; 49	49.10; 50	48.95; 50
Cantanhede	53; 53	47.59; 50	48.24; 50	48.91; 50	49.53; 50	Pedrógão Grande	NA	49.48; 50	47.40; 49	48.14; 49	49.34; 50
Carregal do Sal	47.15; 48	48.39; 50	47.99; 49	49.29; 50	49.40; 50	Penacova	46.21; 45	48.49; 50	48.23; 50	48.75; 50	49.24; 50
Castanheira de Pêra	NA	NA	48.36; 49	48.77; 50	50.14; 51	Penalva do Castelo	47.38; 49	48.09; 50	48.16; 49	49.17; 50	49.34; 50
Castelo Branco	47.84; 48	47.90; 49	48.48; 50	48.84; 50	49.59; 50	Penamacor	42; 42	47.77; 49	48.59; 50	48.51; 50	50.34; 51
Castro Daire	46; 45	48.18; 48.5	47.87; 49	48.76; 50	49.91; 50	Penedono	46.50; 46.5	49.37; 50	48.50; 50	48.86; 50	46.50; 47
Celorico da Beira	48.10; 48	48.27; 49	48.08; 50	48.64; 50	49.13; 50	Penela	47.71; 48	47.97; 50	48.22; 50	48.48; 50	48.81; 50
Coimbra	48.17; 49	47.58; 49	47.83; 49	48.36; 50	49.05; 50	Pinhel	48.10; 50	48.61; 50	48.40; 49	48.82; 50	49.39; 50
Condeixa-a-Nova	47.16; 49	47.27; 48	47.73; 49	48.33; 49	49.24; 50	Pombal	45; 45	47.79; 49	48.54; 50	48.84; 50	49.65; 50
Covilhã	48.55; 50	47.77; 49	48.47; 50	48.77; 50	49.53; 50	Porto de Mós	48.79; 50	47.41; 48	47.83; 49	48.36; 50	49.85; 50
Estarreja	45.33; 45	48.35; 50	48.57; 50	48.98; 50	49.39; 50	Proença-a-Nova	46.98; 48	46.57; 48	47.73; 49	49.06; 50	49.45; 50
Ferreira do Zêzere	47.24; 50	47.65; 49	48.74; 50	48.84; 50	46.21; 48	Resende	47.21; 48.5	48.06; 50	48.63; 50	48.66; 50	44.74; 45
Figueira da Foz	NA	48.92; 50	48.18; 50	48.61; 50	49.24; 50	Sabugal	46.80; 50	48.74; 50	48.49; 50	49.06; 50	49.68; 50
Figueira de Castelo R	46.71; 46	47.37; 48	48.15; 49	47.98; 49	49.38; 50	Santa Comba Dão	46.09; 46	47.93; 49	48.09; 49	49.04; 50	49.76; 50
Figueiró dos Vinhos	NA	NA	47.47; 48	47.97; 49	49.44; 50	São Pedro do Sul	48.49; 49	47.93; 49	48.38; 50	49.34; 50	49.80; 50
Fornos de Algodres	46.07; 45	47.13; 48	48.40; 49	48.52; 50	49.51; 50	Sátão	47; 49.5	47.98; 50	47.91; 49	48.77; 50	49.42; 50
Fundão	47.15; 48	48.07; 49	48.24; 49	48.95; 50	49.77; 50	Seia	NA	49.44; 50	48.58; 50	49.07; 50	49.74; 50
Góis	47.88; 50	47.95; 50	48.23; 50	48.62; 50	49.40; 50	Sernancelhe	48.70; 50	48.39; 49	48.94; 50	49.26; 50	46.76; 47
Gouveia	47.97; 50	47.16; 48	47.62; 48	48.79; 50	49.59; 50	Sertã	47.28; 50	47.26; 49	48.17; 49	48.98; 50	49.94; 51
Guarda	48.30; 50	48.85; 50	48.90; 50	49.09; 50	49.83; 50	Sever do Vouga	NA	48.19; 49.5	48.17; 50	48.45; 50	49.37; 50
Idanha-a-Nova	47.07; 49	47.73; 49	48.01; 49	49.13; 50	49.91; 50	Soure	47.03; 47	47.86; 49	48.03; 49	48.32; 49	49.34; 50
Álhavo	45.43; 45	48.47; 50	48.09; 49	48.27; 49	49.08; 50	Tábua	49; 50	47.50; 48	48.22; 50	48.48; 50	49.05; 50
Lamego	46.74; 47	47.94; 49	48.14; 49	48.19; 49	45.70; 47	Tomar	47.66; 50	47.57; 49	48.12; 49	48.42; 50	47.07; 48
Leiria	48.35; 50	48.33; 50	48.02; 49	48.68; 50	49.40; 50	Tondela	47.65; 49	47.38; 49	48.25; 50	49.01; 50	49.64; 50
Lousã	46.68; 48	47.41; 48	48.12; 49	48.11; 49	48.76; 50	Trancoso	48.98; 49	48.45; 50	48.78; 50	49.09; 50	49.70; 50
Mação	NA	NA	47.83; 49	48.81; 50	48.10; 49	Vagos	48.56; 50	47.87; 50	48.16; 50	48.38; 50	49.35; 50
Mangualde	47.33; 47.5	47.82; 50	48.57; 50	48.89; 50	49.55; 50	Vila de Rei	48.27; 50	48.35; 49	48.94; 50	49.11; 50	49.99; 50
Manteigas	43.71; 45	47.55; 48	48.25; 50	48.45; 49	49.44; 50	Vila Nova de Foz Coa	47.21; 50	47.09; 48	47.49; 48	47.85; 49	49.09; 50
Marinha Grande	46.67; 48	48.11; 49	48.02; 49	48.57; 50	49.36; 50	Vila Nova de Paiva	47.21; 48	47.58; 48	47.96; 49	48.43; 50	49.40; 50
Mealhada	45.73; 44	47.50; 49	47.64; 49	48.18; 50	49.11; 50	Vila Nova de Poiares	46.21; 45.5	47.89; 49	48.12; 49	48.27; 49.5	49.86; 50
Meda	46.92; 49.5	47.79; 49	48.86; 50	48.38; 50	49.78; 50	Vila Velha de Rodão	45.89; 45	47.20; 47	48.16; 49	49.46; 50	49.54; 50
Mira	47.03; 48	47.55; 49	48.74; 50	48.63; 50	49.33; 50	Viseu	47.32; 49	47.95; 49	48.62; 50	49.02; 50	49.52; 50
Miranda do Corvo	46.95; 48.5	47.89; 48	47.75; 48	48.43; 49	49.38; 50	Vouzela	47.57; 48	48.08; 50	48.82; 50	49.11; 50	50.04; 50
Moimenta da Beira	48.47; 50	48.32; 50	48.49; 50	49.27; 50	46.83; 48

**Table 5:** Percentage $(\times 100 \%)$ of women in the data set with an observed menopause per municipality since 1910 to 1960 (Descriptive statistics).

Period (birth year)						Period (birth year)					
Municipality	10–20	21–30	31–40	41–50	51–60	Municipality	10–20	21–30	31–40	41–50	51–60
Águeda	1.00	0.99	0.99	0.98	0.92	Montemor-o-Velho	NA	1.00	1.00	0.99	0.94
Aguiar da Beira	0.99	0.99	0.98	0.97	0.94	Mortágua	1.00	1.00	1.00	0.95	0.91
Albergaria-a-Velha	NA	0.96	0.99	0.98	0.92	Murtosa	NA	1.00	0.99	0.98	0.91
Alcobaça	1.00	0.99	0.99	0.96	0.52	Nazaré	1.00	0.97	0.98	0.93	0.52
Almeida	1.00	1.00	0.99	0.97	0.91	Nelas	1.00	1.00	1.00	0.97	0.93
Alvaiázere	1.00	1.00	0.99	0.96	0.93	Oleiros	0.99	0.99	0.99	0.98	0.89
Anadia	0.98	0.99	0.99	0.97	0.91	Oliveira de Frades	0.99	1.00	0.99	0.95	0.90
Ansião	1.00	0.97	0.99	0.98	0.91	Oliveira do Bairro	1.00	1.00	0.99	0.96	0.90
Arganil	1.00	0.99	0.99	0.97	0.91	Oliveira do Hospital	1.00	0.98	0.99	0.96	0.90
Aveiro	NA	1.00	1.00	0.98	0.90	Ourém	NA	1.00	0.99	0.97	0.43
Batalha	1.00	1.00	1.00	0.97	0.93	Ovar	1.00	1.00	0.99	0.98	0.92
Belmonte	1.00	1.00	1.00	0.98	0.93	Pampilhosa da Serra	NA	NA	0.98	1.00	0.93
Cantanhede	1.00	1.00	1.00	0.98	0.91	Pedrógão Grande	NA	1.00	1.00	0.98	0.93
Carregal do Sal	1.00	1.00	0.99	0.96	0.91	Penacova	1.00	1.00	1.00	0.98	0.90
Castanheira de Pêra	NA	NA	0.98	0.99	0.90	Penalva do Castelo	0.94	0.99	0.99	0.97	0.91
Castelo Branco	1.00	1.00	0.99	0.97	0.92	Penamacor	1.00	1.00	1.00	0.99	0.91
Castro Daire	1.00	1.00	1.00	0.97	0.93	Penedono	1.00	1.00	1.00	0.93	0.40
Celorico da Beira	1.00	1.00	1.00	0.97	0.90	Penela	0.96	0.97	0.99	0.97	0.93
Coimbra	1.00	1.00	1.00	0.98	0.91	Pinhel	1.00	1.00	0.99	0.95	0.90
Condeixa-a-Nova	1.00	0.99	0.97	0.90	0.90	Pombal	1.00	0.99	1.00	0.99	0.93
Covilhã	1.00	0.99	1.00	0.98	0.91	Porto de Mós	1.00	0.99	0.99	0.96	0.91
Estarreja	1.00	1.00	0.99	0.99	0.92	Proença-a-Nova	1.00	1.00	0.99	0.98	0.92
Ferreira do Zêzere	1.00	1.00	1.00	0.92	0.39	Resende	1.00	1.00	0.99	0.77	0.20
Figueira da Foz	NA	1.00	1.00	0.99	0.93	Sabugal	1.00	1.00	1.00	0.96	0.91
Figueira de Castelo R	1.00	1.00	0.99	0.95	0.93	Santa Comba Dão	0.93	1.00	0.99	0.97	0.89
Figueiró dos Vinhos	NA	NA	1.00	0.99	0.89	São Pedro do Sul	1.00	1.00	0.99	0.96	0.92
Fornos de Algodres	0.98	1.00	0.99	0.95	0.90	Sátão	1.00	1.00	1.00	0.98	0.93
Fundão	0.99	1.00	0.99	0.97	0.93	Seia	NA	0.99	0.99	0.97	0.92
Góis	1.00	1.00	0.98	0.96	0.87	Sernancelhe	1.00	1.00	0.99	0.95	0.39
Gouveia	0.99	1.00	0.98	0.94	0.90	Sertã	1.00	0.99	0.99	0.97	0.90
Guarda	1.00	1.00	1.00	0.98	0.93	Sever do Vouga	NA	0.93	0.99	0.98	0.94
Idanha-a-Nova	1.00	1.00	0.99	0.97	0.91	Soure	1.00	1.00	1.00	0.98	0.95
Álhavo	1.00	1.00	1.00	0.98	0.91	Tábua	1.00	0.99	1.00	0.98	0.93
Lamego	0.98	1.00	0.99	0.90	0.32	Tomar	0.99	0.98	0.99	0.96	0.56
Leiria	0.98	0.97	0.99	0.98	0.92	Tondela	0.99	1.00	0.99	0.96	0.92
Lousã	1.00	1.00	1.00	0.98	0.93	Trancoso	1.00	1.00	1.00	0.97	0.90
Mação	NA	NA	0.92	0.97	0.54	Vagos	0.96	1.00	1.00	0.98	0.90
Mangualde	1.00	1.00	1.00	0.98	0.91	Vila de Rei	1.00	1.00	1.00	0.98	0.90
Manteigas	1.00	1.00	0.99	0.96	0.90	Vila Nova de Foz Coa	1.00	1.00	1.00	0.98	0.90
Marinha Grande	1.00	0.99	1.00	0.97	0.91	Vila Nova de Paiva	1.00	1.00	0.99	0.95	0.90
Mealhada	0.92	0.98	1.00	0.98	0.93	Vila Nova de Poiares	1.00	1.00	1.00	0.96	0.93
Meda	1.00	1.00	1.00	0.98	0.92	Vila Velha de Rodão	1.00	1.00	1.00	0.98	0.92
Mira	0.99	1.00	0.99	0.94	0.89	Viseu	0.98	1.00	0.99	0.97	0.92
Miranda do Corvo	1.00	1.00	0.99	0.98	0.91	Vouzela	1.00	0.99	0.99	0.96	0.89
Moimenta da Beira	1.00	1.00	0.99	0.95	0.45						

## Data Availability

The datasets used and/or analyzed during the current study are available from the corresponding author on reasonable request.
